# Role of cardiovascular magnetic resonance in the prognosis of patients with myocardial infarction with non-obstructive coronary arteries

**DOI:** 10.1186/s12968-021-00773-w

**Published:** 2021-07-01

**Authors:** Nuria Vicente-Ibarra, Eloisa Feliu, Vicente Bertomeu-Martínez, Pedro Cano-Vivar, Pilar Carrillo-Sáez, Pedro Morillas, Juan Miguel Ruiz-Nodar

**Affiliations:** 1 Cardiology Service, Elche University Hospital, Alicante, Spain; 2 Magnetic Resonance Imaging Unit, Inscanner S.L. General University Hospital of Alicante, Alicante, Spain; 3 Cardiology Service, San Juan General University Hospital, Alicante, Spain; 4 Cardiology Service. Santa Lucía General University Hospital. Cartagena, Murcia, Spain; 5 Instituto de Investigación Sanitaria y Biomédica de Alicante (ISABIAL), General University Hospital of Alicante, Alicante, Spain; 6 Department of Cardiology, General University Hospital of Alicante, Alicante, Spain

**Keywords:** Myocardial infarction with no obstructive coronary arteries, Cardiovascular magnetic resonance, Adverse cardiovascular events, Myocardial infarction

## Abstract

**Background:**

It is estimated that 5% to 10% of patients with myocardial infarction (MI) present with no obstructive coronary artery lesions. Until now, most studies have focused on acute coronary syndrome, including different clinical entities with a similar presentation encompassed under the term MINOCA (MI with non-obstructive coronary arteries). The aim of this study is to assess the prognosis of patients diagnosed with true infarction, confirmed by cardiovascular magnetic resonance (CMR), in the absence of significant coronary lesions.

**Methods:**

Prospective multicenter registry study, including 120 consecutive patients with a CMR-confirmed MI without obstructive coronary artery lesions. The primary clinical outcome was major adverse cardiovascular events (MACE: death, non-fatal infarction, stroke, or cardiac readmission), assessed over three years.

**Results:**

Seventy-six patients (63.3%) were admitted with a diagnosis of acute coronary syndrome, and 44 (36.6%) for other causes (mainly heart failure); the definitive diagnosis was established by CMR. Most patients (64.2%) were men, and the mean age was 58.8 ± 13.5 years. Patients presented with small infarcts: 83 (69.1%) showed late gadolinium enhancement (LGE) in one or two myocardial segments, mainly transmural (in 77.5% of patients) and with a preserved left ventricular ejection fraction (median 54.8%, interquartile range 37–62). The most frequent infarct location was inferolateral (n = 38, 31.7%). During follow-up, 43 patients (35.8%) experienced a MACE, including 9 (7.5%) who died. In multivariable analysis, LGE in two versus one myocardial segment doubled the risk of adverse cardiac events (hazard ratio [HR] 2.32, 95% confidence interval [CI] 0.97–5.83, p = 0.058). Involvement of three or more myocardial segments almost tripled the risk (HR 2.71, 95% CI 1.04–7.04, p = 0.040 respectively).

**Conclusions:**

Patients with true MI but without significant coronary artery lesions predominantly had small infarcts. Myocardial 3-segment LGE involvement is associated with a significantly higher risk of adverse cardiac events.

## Background

The main physiopathological mechanism underlying acute coronary syndrome (ACS) is local and systemic inflammation, which provokes the rupture of an atheromatous plaque and subsequent coronary thrombosis [1]. Although ACS is generally associated with obstructive coronary artery disease, in up to 30% of these patients neither plaque nor thrombosis are visible in the coronary angiography [2]. This situation has occurred more frequently in recent years, in large part due to increased access to coronary angiography and the existence of more sensitive and specific troponins for diagnosing myocardial infarction (MI) [3]. These advances have led to the definition of a new entity, MI with non-obstructive coronary arteries (MINOCA), whose diagnosis is established when the coronarography shows the following features: (a) it meets universal criteria for MI; (b) the coronary angiogram shows no obstruction of the coronary arteries, defined as the absence of coronary diameter stenosis > 50% on any artery that is potentially responsible for the MI; and (c) there is no specific or overt clinical cause for acute presentation [4]. There are also non-ischemic or non-thrombotic clinical entities that can present with a similar clinical profile to myocardial infarction, such as myocarditis or Takotsubo syndrome. As their prognostic and therapeutic management are different, it is vital to reach an accurate diagnosis in these patients [5].

Cardiovascular magnetic resonance (CMR), especially when employing late gadolinium enhancement (LGE) techniques, is a highly sensitive, non-invasive imaging modality for detecting alterations of the myocardium, allowing identification of the etiology in 65% to 90% of cases with MINOCA [5–7]. Gadolinium is a low molecular weight compound that easily penetrates the capillary pores and spreads throughout the extracellular space. In a normal myocardium, it is washed out in a short period of time, whereas it builds up in the case of myocardial necrosis. The LGE study applies an inversion time, which nulls the signal from the normal myocardium (it appears hypoenhanced). This reveals the hyperenhanced myocardial necrosis with very high sensitivity. CMR is the only imaging technique capable of detecting small foci of fibrosis.

Several investigators have used CMR to analyze diverse cohorts of patients with ACS but without coronary obstruction in the angiographic study [5–19]. The results are not neatly concordant; most cases are considered myocarditis, while only a fraction are true infarctions. Therefore, the prognosis of patients diagnosed with MINOCA and having true MIs (free of the influence of non-ischemic entities) is not well defined. Moreover, the role of CMR for evaluating the prognosis of these patients is also unknown.

The primary aim of this study is to assess the medium-term prognosis of patients with CMR-confirmed MI but without significant lesions on coronary angiography. Moreover, we investigate the morphological characterization of this type of MI on CMR and its relationship with adverse cardiac events during follow-up.

## Methods

This was a multicenter descriptive, prospective study, with a small proportion of retrospectively included patients. We included consecutive patients from our CMR unit, referred from eight centers from May 2009 to June 2017, either during admission for an acute cardiologic event (ACS, heart failure, or ventricular tachycardia) or soon after discharge, when an invasive coronary angiography showed no significant coronary artery lesions. Patients were followed for a median of 2.9 years.

Exclusion criteria were: history of ischemic cardiomyopathy, regardless of revascularization; coronary diameter stenosis of > 50% on the conventional angiography; or midwall/subepicardial LGE with preserved endomyocardium (suggestive of myocarditis or other cardiomyopathies) or its absence on the LGE images.

**Fig. 1:**
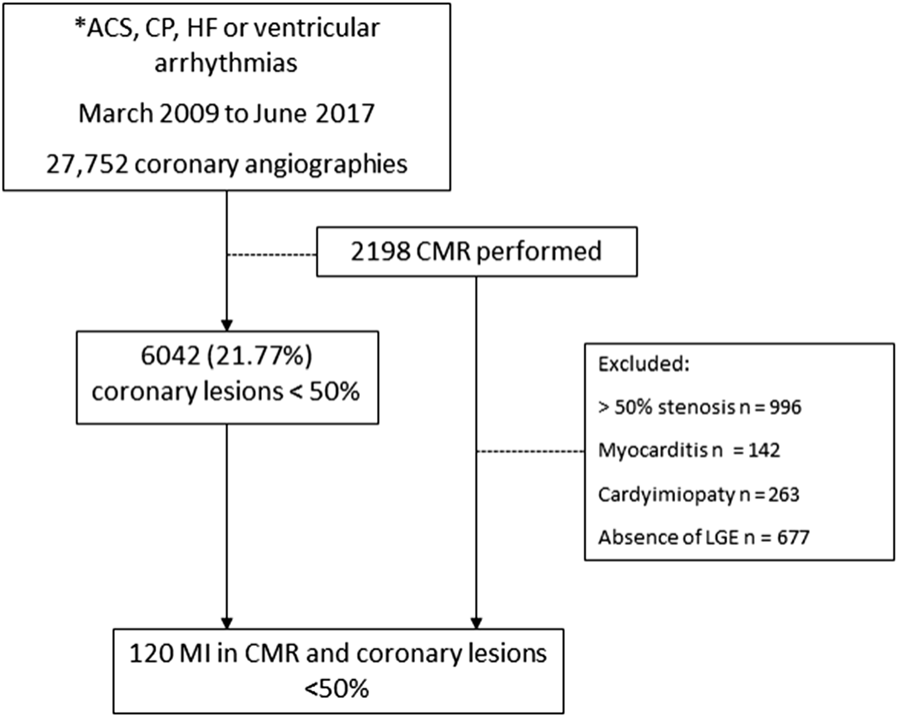
Patient inclusion flowchart. *ACS acute coronary syndrome; *CP* chest pain; *HF* acute heart failure; *CMR* cardiovascular magnetic resonance; *MI* myocardial infarction; *LGE*, late gadolinium enhancement

During the study period, 27,752 coronarographies were performed in patients who had an initial diagnosis of ACS, chest pain, heart failure, or ventricular arrhythmia. CMRs were performed in 2198 of these patients, as indicated by the attending physician. The final study cohort consisted of 120 patients with subendocardial or transmural LGE, compatible with a defined coronary territory and absence of coronary artery diameter stenosis > 50% on angiography. Coronary artery angiograms were performed in three different centers. Studies were additionally reviewed in each one of those centers by another expert interventional cardiologist different from the one who had performed and reported the study. The patient flow chart is shown in Fig. 1.

Clinical and epidemiological variables as well as data related to the coronary angiography and post-discharge treatment were collected. The attending physicians decided on the drug treatment and disease management strategy. Investigators completed follow-up phone interviews and reviews of patients’ clinical records in 99.2% of the cases.

The estimated glomerular filtration rate (eGFR) was calculated using the Modification of Diet in Renal Disease (MDRD) Study equation. All coronary angiograms were reviewed by a second expert interventional cardiologist. The study protocol complied with the Declaration of Helsinki and was approved by the research ethics committee of the reference hospital. All patients provided written informed consent.

### Endpoints

The primary endpoint was the appearance of major adverse cardiovascular events (MACE) at follow-up, defined as death from any cause, non-fatal infarction, stroke, or cardiac readmission.

### CMR protocol

CMR studies were performed using a 1.5 T CMR scanner (Intera, Philips Healthcare, Best, the Netherlands) and a multichannel phased-array antenna devoted to cardiac studies. All images were acquired during breath-holds and were electrocardiogram (ECG)-triggered. The following sequences were performed:

1. Cine Imaging (balanced steady-state free precession [bSSFP]) with at least 20 phases per cardiac cycle (8 mm slice thickness plus 2 mm gap; repetition time/echo time 3.3/1.65 ms; flip angle 60°; matrix 256 × 220). The following planes were obtained: two chamber, four chamber, three chamber and short axis with full left ventricle (LV) coverage from the mitral valve plane to the apex.
2. T2-STIR (short time inversion recovery) black blood sequence turbo spin echo [TSE] on short axis plane (slice thickness: 8 mm plus 2 mm gap, repetition time 2 × R to R interval (RR), echo time 100 ms, matrix 256 × 256).
3. LGE: T1-enhanced, 3-dimensional inversion recovery turbo gradient echo sequence (T1-turbo field echo, repetition time/echo time 4.0/1.24 ms; flip angle 15°; REC voxel MPS 1.5 × 1.5 mm), 10 min after administration of 0.1 mmol/kg of gadobutrol (Gadovist, Bayer Healthcare, Berlin, Germany) on two-chamber, four-chamber, and short axis planes (again covering the entire myocardium). The inversion time was adjusted individually to null the healthy myocardium, oscillating from 250 to 300 ms.

### CMR analysis

One expert CMR radiologist performed the image analysis on an independent workstation provided by the manufacturer (View-Forum release 6.3, Philips Healthcare). In cine imaging, LV ejection fraction (LVEF) (%), end-diastolic volume (LVEDV) and end-systolic (ml/m^2^) volume (LVESV), as well as LV mass (g/m^2^) were calculated by semiautomatic planimetry of the endocardial and epicardial borders on all short-axis views. Also, right ventricle (RV) ejection fraction (RVEF) (%), end-diastolic volume (RVEDV), and end-systolic (ml/m^2^) volume (RVESV) were obtained, again by planimetry of the endocardial border on all short-axis views. A 17-segment model of the heart was used to visually determine the presence of either myocardial edema on T2-STIR weighted-images, or contrast enhancement on LGE images. When myocardial edema and LGE coincided, the finding was considered to be related to the acute onset. The inter and intra-observer variability for the quantification of all exposed CMR parameters in our unit has previously been determined as less than 5%.

### Statistical analysis

Data were entered onto an Excel spreadsheet (Microsoft Corporation, Redmond, Washington, USA) and exported to Stata (version 13.1, StataCorp; College Station, Texas, USA). Categorical variables were expressed as absolute and relative frequencies, while continuous variables were assessed for normality using the Shapiro–Wilk test and expressed as mean and standard deviation (SD) or median and interquartile range (IQR), as appropriate.

Mortality and MACE observed during follow-up were expressed as percentages. Initially, a univariable analysis was performed to test the association between cardiovascular disease risk factors and comorbidity, analytical data and post-discharge treatment. Then a multivariable Cox regression was used to assess the relationship between predictor variables and cardiovascular events; the final model included all variables yielding a p value under 0.05 in univariable analysis, along with any other clinical variables that could plausibly influence the outcome, such as age, sex and different medical treatments upon discharge.

## Results

Patients had a mean age of 58.8 years, and 64.2% were men. Half (51.7%) were hypertensive, while just 16.7% were diabetic. The main reason for ordering a coronary angiography was ACS (n = 76, 63.3%), followed by heart failure (n = 37, 30.8%) and ventricular arrhythmias (n = 7, 5.8%). Table 1 shows patients’ demographic and baseline clinical characteristics.

**Table 1:** Baseline subject characteristics (N = 120)

Variable	n (%)^a^
Age (years), mean ± SD	58.8 ± 13.5
Women	43 (35.8)
Clinical presentation	
ST-elevation ACS	23 (19.2)
Non-ST elevation ACS	53 (44.2)
Dilated cardiomyopathy and/or heart failure	37 (30.8)
Ventricular arrhythmias	7 (5.8)
Electrocardiogram	
Sinus rhythm	103 (85.8)
Atrial fibrillation	17 (14.2)
ST elevation ≥ 2 leads or LBBB	29 (24.2)
Non-ST elevation	64 (53.3)
Comorbidities	
Hypertension	62 (51.7)
Diabetes mellitus	20 (16.7)
Dyslipidemia	40 (33.3)
Current smoking habit	42 (35)
History of atrial fibrillation	17 (14.2)
History of stroke/TIA	7 (5.8)
Alcohol abuse	19 (15.8)
Drug abuse	12 (10)
eGFR (mL/min/1.73 m^2^), mean ± SD	80 ± 34
Treatment at discharge	
Aspirin	72 (60)
Dual antiplatelet therapy	22 (18.3)
Oral anticoagulants	38 (31.7)
Beta-blockers	68 (56.7)
ACEI/ARB	81 (67.5)
Statins	71 (59.2)

*ACS* acute coronary syndrome; *LBBB* left bundle branch block; *TIA* transient ischaemic attack; *eGFR* estimated glomerular filtration rate; *ACEI* angiotensin converting enzyme inhibitor; *ARB* angiotensin II receptor blocker; *SD* standard deviation

^a^Unless noted otherwise as mean ± SD

A high proportion of patients presented arrhythmias at high thrombotic risk; 17 patients (14.2%) had a history of atrial fibrillation (AF). Another 12 patients (10%) were diagnosed with AF during their hospital stay, and 8 (6.7%) developed this condition during follow-up. All told, 11 patients (9.2%) presented an intraventricular thrombus*.*

On the angiography, 63.3% of the patients had angiographically normal coronary arteries, while 21.7% were described as having parietal irregularities or coronary artery ectasia, and 15% had non-significant (< 50% obstruction) atherosclerotic lesions. Thirty-three patients (27.5%) underwent a ventriculography, and 65% presented segmental contractility abnormalities (Table 2).

**Table 2:** Diagnostic imaging results

Coronary angiography, n (%)	
Normal	76 (63.3)
Parietal irregularities	25 (20.9)
Non-obstructed coronary lesions	18 (15)
Ectatic coronary arteries	1 (0.8)
Ventriculography	33 (27.5)
Cardiovascular magnetic resonance	
LVEDVI (mL/m^2^), median (IQR)	89.2 (70.8–119.2)
LVESVI (mL/m^2^), median (IQR)	40 (26.7–70.7)
LVEF (%), median (IQR)	54.8 (37–62)
RVEDVI (mL/m^2^), median (IQR)	70.1 (57.1–81.6)
RVESVI (mL/m^2^), median (IQR)	22.3 (17.8–35)
RVEF (%), median (IQR)	67 (57–68.9)
LV mass index (g/m2), median (IQR)	97.2 (83.8–157.6)
Presence of late gadolinium enhancement, n (%)	
Subendocardial	19 (15.8)
Transmural	93 (77.5)
Both	8 (6.7)
Myocardial segments with enhancement, n (%)	
1 segment	40 (33.3)
2 segments	43 (35.8)
≥ 3 segments	37 (30.9)
Main infarct locations, n (%)	
Inferolateral	38 (31.7)
2 affected myocardial territories	9 (7.5)
Right ventricle	3 (2.5)

**Fig. 2:**
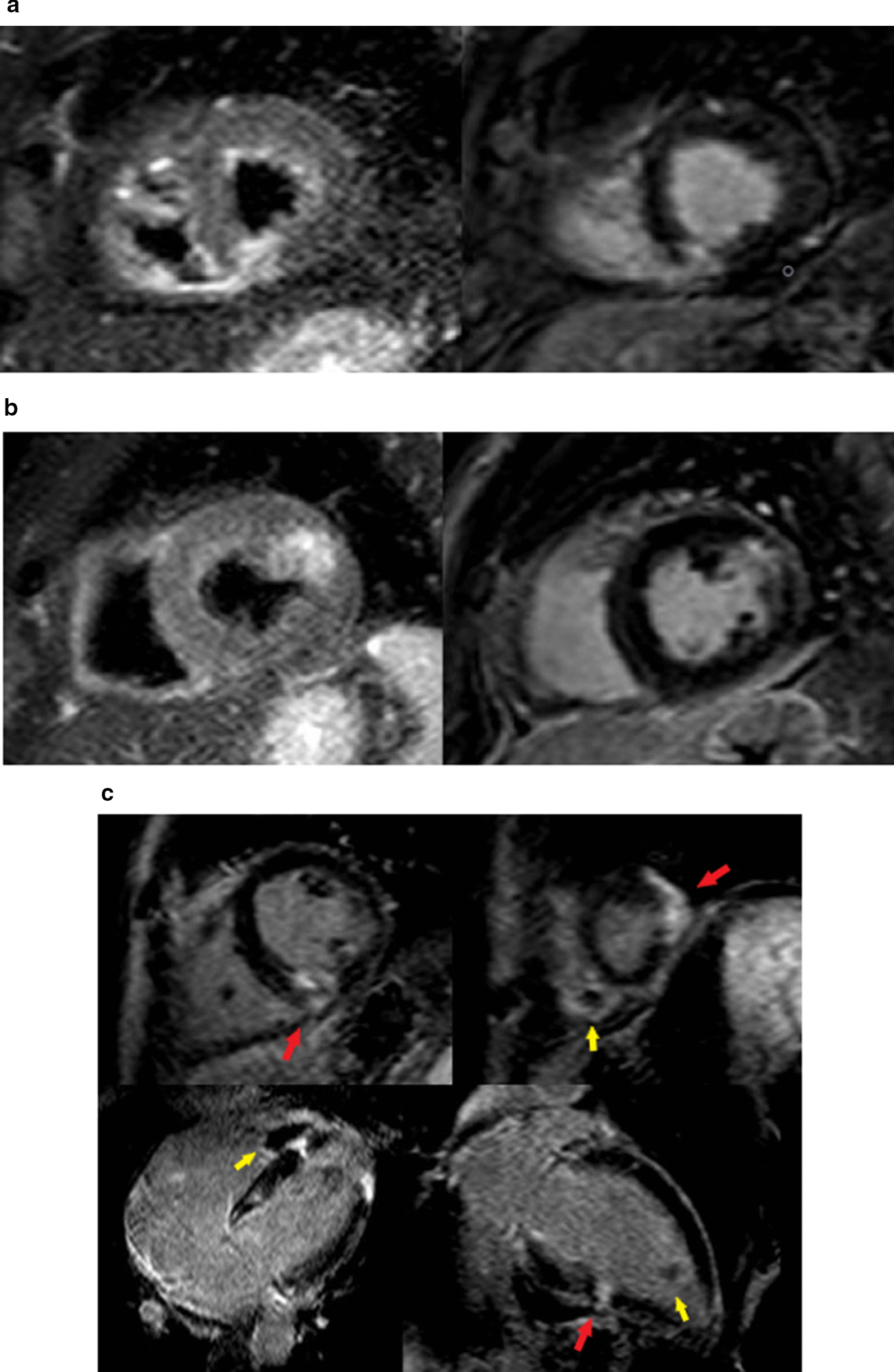
CMR images. **a** Focal transmural inferoseptal acute myocardial infarction. Left: T2 short tau inversion recovery (STIR) short-axis image showing focal transmural edema. Right: Late gadolinium enhancement (LGE) CMR short-axis corresponding image showing transmural hyperenhancement. **b** Anterolateral subendocardial acute myocardial infarction. Left: T2 STIR short-axis image showing focal subendocardial edema. LGE short-axis corresponding image showing focal subendocardial hyperenhancement. **c** Presence of three distinct focal myocardial infarctions: lateroapical, septomedial and inferomedial (red arrows), with images suggestive of thrombus in both ventricles (yellow arrows). LGE images (upper: 2 short-axis views; lower: 4-chamber and 2 chamber views) showing the 3 foci of enhancement. *LGE* late gadolinium enhancement

*LVEDVI* left ventricular end-diastolic volume index; *LVESVI* left ventricular end-systolic volume index; *LVEF* left ventricular ejection fraction; *RVEDVI* right ventricular end-diastolic volume index; *RVESVI* right ventricular end-systolic volume index; *RVEF* right ventricular ejection fraction;

The CMR was performed at a median of 12 days. LGE demonstrated transmural enhancement in 93 cases (77.5%), and subendocardial enhancement in 19 (15.8%). Eight patients, (6.7%) had both types in two distinct regions (Fig. 2). Almost three-quarters of the patients (69.8%) presented with a small infarction on LGE, defined as enhancement in one or two LV segments. The most frequent infarct location was the inferolateral wall (Table 2; Fig. 2).

In the analysis of treatment upon discharge, we observed an underuse of all drugs recommended to treat ischemic cardiomyopathy; only 67.5% of patients were discharged with a prescription for angiotensin converting-enzyme inhibitors (ACEI) or angiotensin II receptor blockers (ARBs); 56.7%, beta-blockers; 59.2%, statins; and 60%, acetylsalicylic acid. Only a fifth of the patients received dual anti-platelet therapy on discharge (Table 1).

### Follow-up

Complete follow-up was achieved in 99.2% of the patients, for a median period of 2.9 (IQR 1.5–4.8) years. Nine patients (7.5%) died—2 from cardiovascular causes—and 7 (5.8%) presented a new non-fatal myocardial infarction. More than one third (35.8%) had a MACE (Table 3).

**Table 3:** Adverse events during follow-up in patients with true myocardial infarctions and coronaries without obstructive lesions

Adverse events, n (%)	All patients	1 segment	2 segments	≥ 3 segments
MACEs	43 (35.8)	8 (20)	19 (44.2)	16 (44.4)
Non-fatal myocardial infarction	7 (5.8)	0 (0)	3 (7)	4 (11.1)
Ischemic stroke	4 (3.3)	1 (2.5)	1(2.3)	2 (5.6)
All-cause mortality	9 (7.5)	4 (10)	3 (7)	2 (5.6)
Cardiovascular death	2 (1.7)	1 (2.5)	0 (0)	1 (2.8)
Cardiovascular readmissions	22 (18.3)	3 (7.5)	12 (27.9)	8 (22.2)
Atrial fibrillation (all)	37 (30.8)	9 (22.5)	18 (41.9)	10 (27)

MACEs: major adverse events (non-fatal myocardial infarction or ischemic stroke or death of any cause or readmissions due to cardiac causes)

**Fig. 3:**
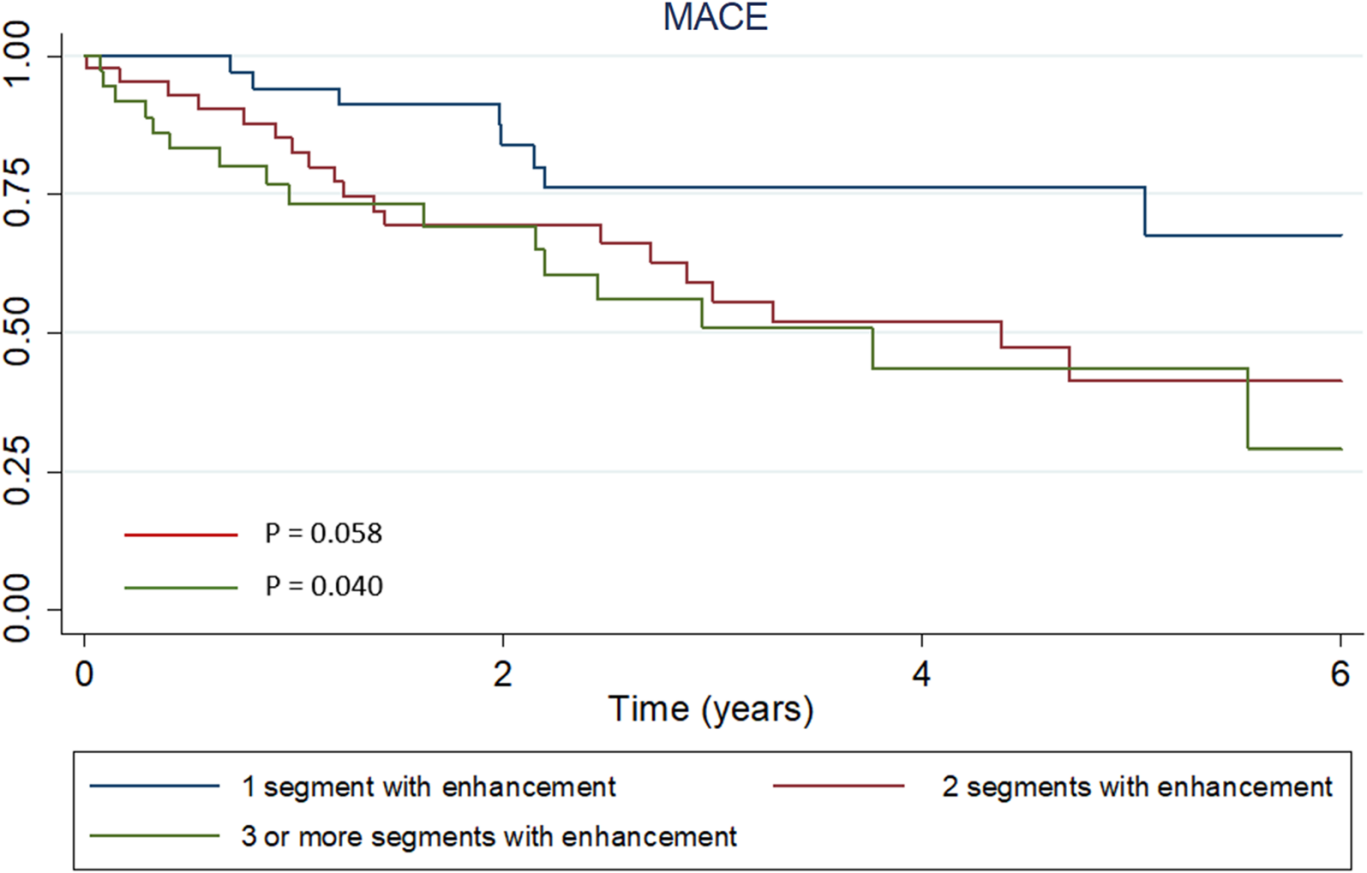
Kaplan–Meier survival curve for MACE depending on the number of segments affected in the CMR. MACE: non-fatal myocardial infarction, ischemic stroke, death from any cause or readmissions due to cardiac causes

In the univariable analysis, low LVEF, LV dilatation, and MI size were associated with a poor prognosis. The involvement of two myocardial segments in CMR was associated with a twofold increased risk of MACE (hazard ratio [HR] 2.26, 95% confidence interval [CI] 0.98–5.19; p = 0.056) without reaching statistical significance; while involvement of three segments or more almost tripled that risk (HR 2.97, 95% CI 1.26–6.95, p = 0.012). The results in univariable analysis remained significant in multivariable analysis (Table 4; Fig. 3).

**Table 4:** Independent predictors of adverse cardiovascular events during follow-up, Cox regression analysis

Risk factors	Univariable analysis HR (95% CI)	p value	Multivariable analysis HR (95% CI)	p value
Age years	1.02 (0.99–1.04)	0.142	1.02 (0.99–1.02)	0.230
Female sex	0.54 (2.27–1.08)	0.080	0.52 (0.24–1.14)	0.104
Clinical presentation^a^				
DCM and/or heart failure	1.25 (0.65–2.43)	0.500		
Ventricular arrhythmias	0.93 (0.32–2.70)	0.901		
Renal insufficiency	2.23 (1.16–4.31)	0.017	2.19 (1.05–4.55)	0.036
LVEF	0.98 (0.96–0.99)	0.027	1.00 (0.97–1.03)	0.781
LVEDVI	1.01 (1.00–1.02)	0.009	1.00 (0.99–1.01)	1.180
Myocardial segments with enhancement^b^				
2 segments	2.26 (0.98–5.19)	0.056	2.32 (0.97–5.83)	0,058
≥ 3 segments	2.97 (1.26–6.95)	0.012	2,71 (1.04–7.04)	0,040
Aspirin	1.32 (0.72–2.44)	0.366		
Dual antiplatelet therapy	0.77 (0.42–1.43)	0.417		
Oral anticoagulants	1.43 (0.77–2.65)	0.257		
Beta-blockers	0.15 (0.62–2.14)	0.663		
ACEI/ARB	1.12 (0.58–2.16)	0.370		
Statins	0.78 (0.42–1.45)	0.436		

*HR* hazard ratio; *CI* confidence interval; *MACE* major adverse cardiovascular event; *DCM* Dilated cardiomyopathy; *LVEF* left ventricular ejection fraction on cardiac magnetic resonance; *LVEDVI* left ventricular end-diastolic volume index on cardiac magnetic resonance; *ACEI* angiotensin converting enzyme inhibitor; *ARB* angiotensin II receptor blokers

^a^Reference: clinical presentation as ACS

^b^Reference: involvement of a single segment of myocardial enhancement

## Discussion

This is the first registry study to assess the role of CMR for evaluating the prognosis of patients with true, CMR-confirmed MI and without significant lesions in the coronary arteries. Thus, we excluded other non-ischemic entities like myocarditis or Takotsubo syndrome. The results suggest that this entity is associated with a high incidence of MACE, including mortality.

To date, published studies have analyzed the clinical characteristics, treatment, and prognosis in patients with MI but without significant coronary lesions, focusing on a heterogeneous group of entities encompassed under MINOCA, including true MI, myocarditis, Takotsubo syndrome, and pulmonary thromboembolism [20]. These entities probably have differential prognoses and characteristics, so outcomes in this clinically diverse population cannot be extrapolated to patients with true MI but without significant lesions. Moreover, infarction in the absence of coronary artery lesions does not always occur in the context of an ACS; it can also present in other clinical scenarios like heart failure or ventricular arrhythmia. Thus, our series includes different forms of presentation in patients with CMR-confirmed MI and coronary artery lesions with obstruction under 50%.

In most published series on MINOCA, patients with an ACS but without significant lesions have been younger and more likely to be women than patients with obstructive lesions [21, 22]. Our sample confirms this tendency, with a younger mean age, a greater proportion of women, and a lower prevalence of risk factors (particularly diabetes) compared to ACS registries in the same population [23–25].

There are various mechanisms that can explain the existence of an infarct without the presence of coronary artery lesions, such as coronary spasm, a repermeabilized thrombus, misinterpretation of angiograms or microvascular dysfunction [26–28], or coronary artery embolization [29, 30]. This last cause could explain a large portion of the MIs in our cohort given the high prevalence of thrombotic arrhythmias (30.8%). Moreover, neurologists pointed to a probable embolic origin for all the strokes occurring during follow-up.

There was a notably low prescription rate for class I drugs indicated in clinical practice guidelines for secondary prevention of events in ischemic cardiomyopathy (aspirin, dual anti-platelet therapy, beta-blockers, ACEI/ARBs, and statins). This is probably attributable to the lower risk profile in these patients and to delays in diagnostic confirmation by CMR. Just 17.5% of the CMR studies were undertaken during the patients’ index hospitalization, which resulted in 74 (61.7%) patients being discharged with a diagnosis other than MI—modified only in follow-up. This brings to mind the most recent European guidelines for ACS with ST-segment elevation, which recommend performing CMR within two weeks of symptoms onset to optimize diagnostic accuracy and enable the best treatment decisions after the event [4]. Likewise, the new European Society of Cardiology guide for ACS without persistent ST-segment elevation includes a recommendation, with level 1b evidence, to perform CMR in all MINOCA patients [31]. Furthermore, a Swedish study [32] assessed drug treatments in major cardiovascular events (infarction, stroke, all-cause mortality, and admission due to heart failure), reporting beneficial effects for statins and ACEI/ARBs, a positive tendency for beta-blockers, and no effect for dual anti-platelet therapy. However, the study population consisted of all patients discharged from hospital with a diagnosis of acute MI and coronary artery stenosis of < 50%. There was no diagnostic confirmation of true infarctions, so other clinical entities with a similar presentation to ACS could have been included, including conditions with very different prognoses and therapeutic management. The underuse of medical treatment in this entity could contribute to the poor medium-term prognosis.

CMR is the most sensitive and specific technique for diagnosing abnormalities in MINOCA. In our series, patients presented with small infarcts: 83 (69.1%) showed LGE in one or two myocardial segments, mainly transmural (in 77.5% of patients) and with a conserved LVEF (median 54.8%, IQR 37–62). The most frequent location of the infarct was inferolateral (n = 38, 31.7%), with segment 14 (apical septal) remaining the least affected. Furthermore, CMR enables specification of the size and location of the MI, among other parameters that are closely related to the prognosis of patients with ischemic cardiomyopathy. In our study, multivariate analysis showed that the involvement of three or more myocardial segments estimated by CMR was associated with a threefold increase risk in adverse cardiac events in comparison with one-segment involvement (HR 2.97, 95% CI 1.26–6.95, p = 0.012). These findings show that CMR is not only a good diagnostic tool; it could also determine the prognosis in patients with true MI but without significant coronary artery stenoses.

Until recently, scientific evidence on the prognosis of patients with ACS but without significant coronary artery stenoses was based only on a few small studies that indicated an excellent prognosis and high 10-year survival rates [33–35]. Emerging evidence shows a worse prognosis, with in-hospital mortality of 0.9% to 4.7% per year [36]. However, all these studies have taken place in the context of ACS, mixing clinical entities whose prognoses are not comparable, which could explain the great variability in the results. Our study, with a median follow-up of 2.9 years, describes a specific population of patients with true infarcts but without significant coronary artery lesions, indicating a poor prognosis. Although two-thirds of the participants had small MIs, the associated mortality was 7.5% and MACE rate was 35.8%. These data are more concordant with the general prognosis of patients with ischemic cardiomyopathy.

### Limitations

Our study has several limitations. One of the limitations of this study is the missing data in baseline characteristics, which is typical of any registry like ours. Moreover, its observational nature makes it impossible to establish a causal relationship, although it does establish associations that can be tested in future studies. The fact that the study was observational also means that it did not influence how clinicians managed patients’ diagnosis and treatment, so CMR may have been underutilized in patients with acute events but no significant lesions. Another limitation is that no intracoronary imaging techniques were used; these may have identified acute ruptures in plaque that were not apparent on the conventional coronarography.

An additional limitation of the study is the existence of pathologies with findings that could be similar in CMR, such as sarcoid or hypereosinophilic syndromes. However, the clinical context, together with analytical data and other complementary tests, led us to the final diagnosis of MINOCA.

The study was in patients with CMR-confirmed MI but without significant coronary artery lesions, including patients with MINOCA but also other forms of presentation, such as ventricular arrhythmias or acute heart failure. We consider this to be the greatest strength of our study, as we included a population (CMR-diagnosed infarction) that has not been specifically studied up to now and whose prognosis was heretofore unknown.

## Conclusions

Patients with CMR-confirmed true MI and without significant coronary artery stenoses have a poor medium-term prognosis, with a high incidence of cardiovascular events and mortality. This type of MI is predominantly small. Involvement of three or more myocardial segments is associated with a significantly higher risk of adverse cardiac events. Our study reflects the importance of performing CMR in patients with MINOCA in order to improve the diagnosis of infarction and apply adequate secondary prevention measures.

## Data Availability

The datasets used and/or analyzed during the current study are available from the corresponding author on reasonable request.

## Abbreviations

ACEI: Angiotensin converting enzyme inhibitor
ACS: Acute coronary syndrome
AF: Atrial fibrillation
ARB: Angiotensin receptor blocker
bSSFP: Balanced steady state free precession
CMR: Cardiovascular magnetic resonance
ECG: Electrocardiogram
eGFR: Estimated glomerular filtration rate
LGE: Late gadolinium enhancement
LV: Left venricle/left ventricular
LVEDV: Left ventricular end-diastolic volume
LVEDVI: Left ventricular end-diastolic volume index
LVEF: Left ventricular ejection nfraction
LVESV: Left ventricular end-systolic volume
LVESVI: Left ventricular end-systolic volume index
MACE: Major adverse cardiovascular events
MI: Myocardial infarction
MINOCA: Myocardial infarction with no obstructive coronary arteries
RV: Right ventricle/right ventricular
RVEDV: Right ventricular end-diastolic volume
RVEDVI: Right ventricular end-diastolic volume index
RVEF: Right ventricular ejection fraction
RVESV: Right ventricular end-systolic volume
RVESVI: Right ventricular end-systolic volume index
STIR: Short tau inversion recovery
STIR: Short tau inversion recovery
TSE: Turbo spin echo
